# Empirical Evidence for Species-Specific Export of Fish Naïveté from a No-Take Marine Protected Area in a Coastal Recreational Hook and Line Fishery

**DOI:** 10.1371/journal.pone.0135348

**Published:** 2015-08-14

**Authors:** Josep Alós, Antoni Puiggrós, Carlos Díaz-Gil, Miquel Palmer, Rosario Rosselló, Robert Arlinghaus

**Affiliations:** 1 Department of Biology and Ecology of Fishes, Leibniz-Institute of Freshwater Ecology and Inland Fisheries, Müggelseedamm 310, 12587, Berlin, Germany; 2 Instituto Mediterráneo de Estudios Avanzados, IMEDEA (CSIC-UIB), C/Miquel Marqués 21, 07190, Esporles, Illes Balears, Spain; 3 Laboratori d'Investigacions Marines i Aqüicultura, LIMIA (Balearic Government), C/Eng Gabriel Roca 69, 07157, Port d'Andratx, Illes Balears, Spain; 4 Division of Integrative Fisheries Management, Faculty of Life Sciences and Integrative Research Institute for the Transformation of Human-Environmental Systems, Humboldt-Universität zu Berlin, Invalidenstrasse 42, 10155, Berlin, Germany; Department of Agriculture, AUSTRALIA

## Abstract

No-take marine protected areas (MPAs) are assumed to enhance fisheries catch via the “spillover” effect, where biomass is exported to adjacent exploited areas. Recent studies in spearfishing fisheries suggest that the spillover of gear-naïve individuals from protected to unprotected sites increases catch rates outside the boundaries of MPAs. Whether this is a widespread phenomenon that also holds for other gear types and species is unknown. In this study, we tested if the distance to a Mediterranean MPA predicted the degree of vulnerability to hook and line in four small-bodied coastal fish species. With the assistance of underwater video recording, we investigated the interaction effect of the distance to the boundary of an MPA and species type relative to the latency time to ingest a natural bait, which was considered as a surrogate of fish naïveté or vulnerability to fishing. Vulnerability to angling increased (i.e., latency time decreased) within and near the boundary of an MPA for an intrinsically highly catchable species (*Serranus scriba*), while it remained constant for an intrinsically uncatchable control species (*Chromis chromis*). While all of the individuals of *S*. *scriba* observed within the MPA and surrounding areas were in essence captured by angling gear, only one fifth of individuals in the far locations were captured. This supports the potential for the spillover of gear-naïve and consequently more vulnerable fish from no-take MPAs. Two other species initially characterized as intermediately catchable (*Coris julis* and *Diplodus annularis*) also had a shorter latency time in the vicinity of an MPA, but for these two cases the trend was not statistically significant. Overall, our results suggest that an MPA-induced naïveté effect may not be universal and may be confined to only intrinsically highly catchable fish species. This fact emphasizes the importance of considering the behavioural dimension when predicting the outcomes of MPAs, otherwise the effective contribution may be smaller than predicted for certain highly catchable species such as *S*. *scriba*.

## Introduction

The implementation of marine protected areas (MPAs) is a widespread management tool for the conservation of marine fisheries resources [1–5]. This is because the spatial protection of marine fauna from harvesting in an MPA is thought to have multiple desirable ecological effects. One of the most important effects is the “spillover” of larvae and adult biomass from inside an MPA to the unprotected areas outside [6–10]. Furthermore, the spillover is expected to not only maintain local populations at high abundance near reserves but also to preserve the natural meta-population structure [11–13], which ultimately should contribute to the maintenance of high catches and yields at broader spatial scales [10,14–16]. However, as MPAs are rarely placed at random and the dispersal of pelagic eggs and larvae alongside stochastically varying oceanographic conditions is commonplace in marine environments, conclusively demonstrating the spillover of fish born inside MPAs has proven to be challenging [16–18]. Nevertheless, common wisdom suggests that a properly scaled and positioned MPA should result in biomass accumulation within the reserve, which could benefit the fisheries operating outside of the boundaries [19–21].

The behavioural dimension of fish inhabiting protected areas is a frequently ignored but important contribution to the benefits of an MPA [22]. Specifically, exporting fish with particular behavioural types from an MPA may elevate the catchability of the subpopulation entering the exploited sites [22]. Consistent individual variation in behaviour across multiple exploited fish species (also known as personality or temperament) has been well documented [23,24]. Such consistent behavioural variation has both ecological and genetic causes [25,26] and has been found to be related to vulnerability to fishing gears [27,28]. Moreover, it is well known that species differ consistently in behavioural modes. For example large top predatory fishes exhibit a foraging behaviour that is markedly different from the behaviour of smaller-bodied foraging fishes [29–31]. Species or individuals within populations that take more risks should also be more likely to be captured by a fishing gear [30]. Therefore, it is possible that the protection offered by a MPA may facilitate the spillover of highly catchable species and individuals to surrounding sites.

Evidence of the aforementioned spillover of naïve fish from protected to exploited sites is so far scarce for gears other than spearfishing [22]. In the context of the exploitation of coral reef fishes by spearfishing, it has recently been demonstrated in a series of studies that the vulnerability of individuals exported from MPAs is higher than the vulnerability of individuals found in exploited areas for some species, which supports the idea of the spillover of “naïve fish” [32]. This new concept is defined as the export of highly vulnerable behavioural phenotypes from no-take protected zones to surrounding exploited areas [32,33]. This finding has been recently confirmed in a study by Januchowski-Hartley et al. [34] who demonstrated that for certain species fish that were observed near MPAs allowed a closer approach by divers before initiating a flight response compared to fish in exploited sites. These flight responses are particularly relevant in the context of diving-based spearfishing, and although the reefs studied by Januchowski-Hartley and co-workers were likely also exploited by other gear types, the behavioural responses found most likely were caused by avoidance reactions to divers. Whether the enhanced naïveté to spearfishing of some species and individuals living close to MPAs is a widespread pattern across fisheries systems such as hook and line (angling), is so far unknown.

In a recent study, Alós et al. [30] compared the behaviour of two small-bodied coastal fish in the context of recreational hook and line fishing and revealed a reduction in the vulnerability to angling in *Serranus scriba* at sites with an increased exploitation effort. Hence, the findings presented by Januchowski-Hartley et al. [22] in spearfished coral reef fishes may also extend to recreationally angled fish stocks. However, Alós et al. [30] found a reduction in vulnerability to fishing pressure in only one of the studied fish species, the carnivorous *S*. *scriba*, but not in the omnivorous *Diplodus annularis*. Similarly, the alteration of behaviour of fish observed in response to spearfishing by Januchowsky-Hartley et al. [32,35,36] was not universal across all coral reef species, presumably because some species were not targeted or were behaviourally or physically uncatchable by the local fishing gears. Generally, one should expect species-specific changes in vulnerability and naïveté along a gradient of fishing pressure from an MPA to exploited sites.

In a recreational angling context, exploited fish species should differ in their intrinsic cathability to hook and line gear for two main reasons. First, gape size plays a role in cathability because hook, bait and lure types limit the possibility of ingestion and capture by small-bodied individuals with small gapes [37]. Second, foraging mode and learning abilities play an important role in the catchability. For example, omnivorous fishes feeding on non-mobile prey might be evolutionarily adapted towards a shyer behavioural life-style, with a careful trade-off between foraging opportunities and the risk of predation. However, carnivorous species might be adapted to attack whenever foraging chances occur because their prey is mobile and encounters are less frequent [30]. The result should be a higher intrinsic catchability of carnivorous species when presented with baits offered by anglers compared to omnivorous species exposed to the same baits [30]. In addition, more aggressive, active or bold individuals have been found more likely to be captured by angling gear [27,28,38–41], leaving behind individuals that are intrinsically less vulnerable and harder to catch [42,43]. Therefore, it is expected that the absence of behaviourally-selective exploitation within an MPA promotes individuals with naïve phenotypes in some recreationally exploited species. This naïve biomass may spillover to the surrounding exploited areas, as seems to occur in spear-fished coral reef species [22].

If a change in behaviour is indeed observed in an exploited species, it could be brought about by either the above mentioned harvesting-induced evolution over generations or by learning (both individual and social) to reduce the likelihood of future capture within a generation. The latter mechanism may be particularly pronounced in catch-and-release angling fisheries [44,45], and should contribute to the species-specific export of naïve fish from MPAs. Furthermore, species differ in their learning ability [46], and this is another reason to expect differences in the intrinsic catchability of species. For example, learning to avoid future capture has been demonstrated in piscivorous pike (*Esox lucius*) fished with artificial lures (although there was no learning response with natural baits) [47,48], as well as in omnivorous fishes such as carp (*Cyprinus carpio*) and rainbow trout (*Oncorhynchus mykiss*) [39,44,49]. Any ban of recreational angling inside MPAs should maintain vulnerable, naïve genotypes and phenotypes via selection and learning, respectively. The strength of the effect should be species specific and these fish may spread outside the MPA, creating a species-specific gradient of fish naïveté or vulnerability. Considering this background, we hypothesized that recreational angling around MPAs would benefit from the spillover of naïve fish in selected fish species due to the creation of a naïveté/vulnerability gradient. Our hypothesis should hold particularly true for those species that are intrinsically highly catchable [32]. By contrast, intrinsically highly uncatchable species, which show little variation in vulnerability independent of fishing site [30], should show little or no change in vulnerability to angling in response to spatial fishing pressure.

## Materials and Methods

### Ethics Statement

The study and the deployment of the underwater cameras were authorised by those responsible for marine natural resources, the MPA, and surrounding areas in Palma Bay (Fisheries Department of the Balearic Island) through a permit to the REC2 Project (ref: CTM2011-23835). The study was based on observations made through underwater video recordings of wild animals and did not imply the capture, sacrifice or experiment with animals, nor did it involve endangered or protected species.

### Study site and study species

The study was conducted around an MPA located at Palma Bay (Southwestern part of Mallorca Island, NW Mediterranean; see details of this MPA in Alós and Arlinghaus [19]). The Mediterranean Sea is a hotspot for marine biodiversity, which includes several species that are highly vulnerable to fishing [50]. The increase in the number of MPAs in the Mediterranean over the last two decades to counteract human-related impacts has been notable [51]. Moreover, the general importance of MPAs for ensuring the sustainability of recreational fisheries in the Mediterranean has recently been emphasized [52], increasing the relevance of studying how MPAs and fish behaviour interact to shape spatial gradients in vulnerability. We studied the boat recreational angling (hook and line) fishery over *Posidonia oceanica* seagrass meadows [53]. This highly popular local fishery is characterized by the exploitation of a diverse fish community for food consumption, and it is mainly composed by small-bodied coastal species with small home ranges [54–56].

The three most frequently captured species in the recreational angling fishery are *Diplodus annularis* (Sparidae), *Coris julis* (Labridae) and *Serranus scriba* (Serranidae). All of these species were part of the experimental design presented here and had similar sizes. The different intrinsic catchabilities of these species meant that recreational angling affects the three species in distinct ways. With respect to *S*. *scriba*, using fishery-dependent data (i.e., experimental hook and line angling), Cardona et al. [55] reported a negative relationship between fishing intensity and a number of population metrics (relative abundance, biomass and average size, see also [57]). Furthermore, Alós and Arlinghaus [19] reported a higher abundance of *S*. *scriba* in MPAs compared to nearby areas open to fishing. These findings suggest that recreational angling reduces the numerical abundance of *S*. *scriba*. In addition, recreational angling may alter several key life-history traits in *S*. *scriba*, as angling has caused a shift in resource investment away from growth and towards reproduction, ultimately resulting in the downsizing of adult body length [58]. The high intrinsic catchability of *S*. *scriba* has been attributed to a combination of its high risk-taking behaviour [58] which is common for apex predators (in this case within the seagrass habitat of *P*. *oceanica* ([59,60]), and its large mouth gape relative to body size, which facilitates the intake of baited hooks [61]. These characteristics do not apply to *D*. *annularis* and *C*. *julis*, which are omnivorous, are in lower trophic levels and are consequently less aggressive in their foraging behaviour. Further, the smaller ratio between the mouth gape and body size of these two species makes them intrinsically less catchable to baited hooks for physical reasons [59,60]. There is also no evidence that recreational angling substantially depletes the abundance or biomass of these two species when comparing exploited and unexploited sites [19,55], which suggests that they either compensate for fishing mortality better than *S*. *scriba* or are simply less catchable. Therefore, *D*. *annularis* and *C*. *julis* can be considered intrinsically less catchable than *S*. *scriba*. We also considered the case of *Chromis chromis* (Pomacentridae) as a largely un-harvested control. *C*. *chromis* is an omnivorous micro-feeder and it is only occasionally captured by recreational anglers [53]. This species is abundant within seagrass habitats and therefore constitutes an appropriate control species.

**Fig 1:**
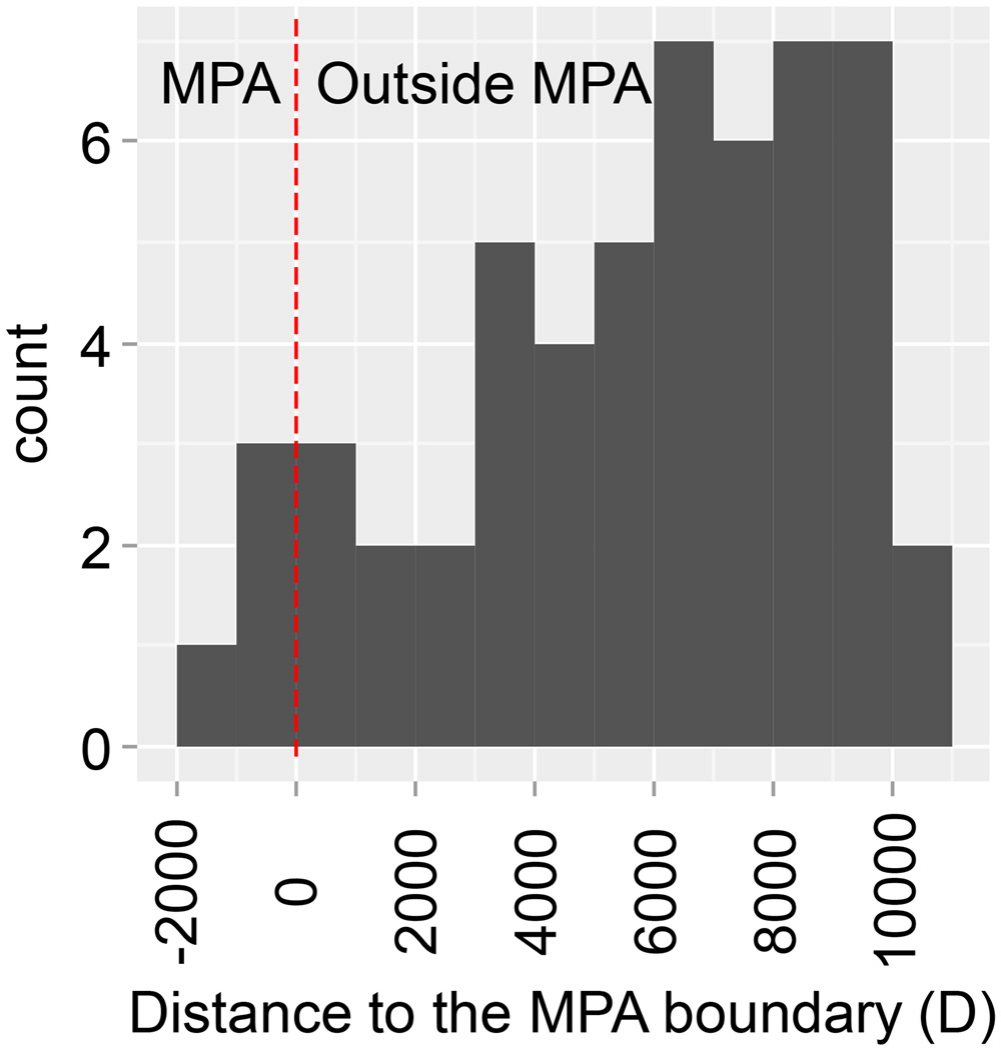
Histogram of distances to the boundary of the marine protected area (MPA) of the Palma Bay (Mallorca Island, NW Mediterranean Sea) of the study site locations (n = 54) where the underwater video cameras were deployed to measure the latency times. We plotted the one-dimensional distribution of the distances considered in the study. Note that negative distances from the boundary are located inside the no-take MPA, minimum values are -1,042 m and maximum distance 10,649 m away from the MPA’s boundary.

The study area spans the MPA of Palma Bay and surrounding areas (Fig 1). This MPA extends from the coastline to the 30 m isobath and creates a protected area for fish inhabiting the *P*. *oceanica* seagrass of Palma Bay. All fishing activities have been regulated since 1982, albeit they were not enforced until the late 1990s (http://dgpesca.caib.es). The MPA is composed of two areas with different levels of protection: (1) the sanctuary area (~2 km^2^), where all fishing activities are forbidden, and (2) a buffer area where both artisanal and recreational fishing are regulated by temporal closures, rod-limits, bag limits and minimum-size limits. We randomly generated 54 sampling sites both within and around the no-take MPA. These sites were constrained to be over seagrass meadows and set at an increasing distance from the centre of the MPA (Fig 1). The distance from the sampling points to the boundary of the no-take MPA ranged from -1,042 m (located inside the no-take MPA) to 10,649 m (Fig 1). The area surrounding the MPA is highly frequented by local anglers as described in Alós and Arlinghaus [19].

### Measuring naïveté in the field

**Fig 2:**
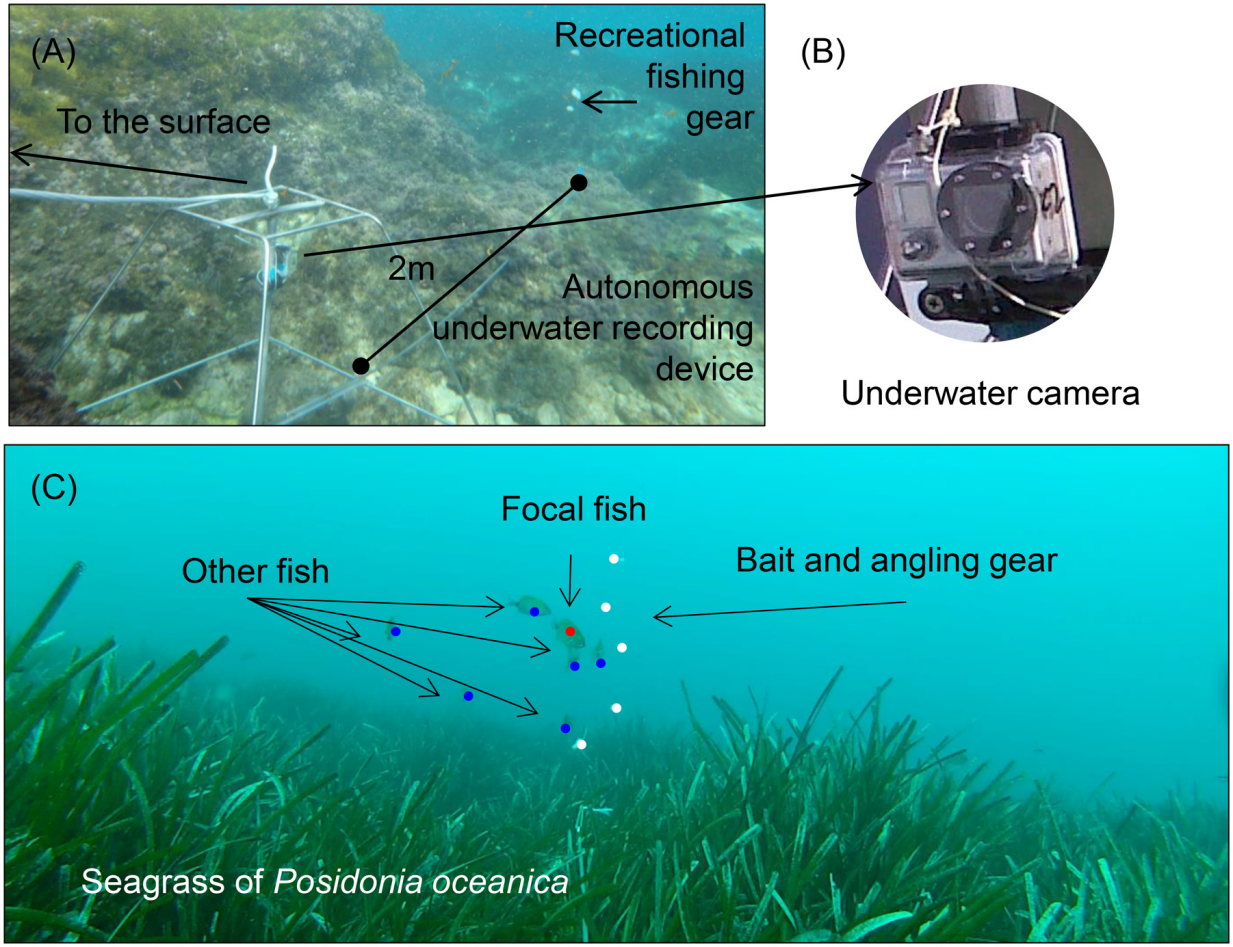
Description of the device used to measure latency time of marine coastal fish targeted by recreational anglers. The device was composed of a structure of stainless steel, the camera and a dipstick of 2 m length to which the fishing gear was attached (panel A). Panel B shows the underwater camera used (Go Pro Inc., 2014). Panel C shows a scene of one of the videos obtained. The focal fish (i.e., the fish for which the latency time was measured) is indicated in red. Blue points indicate the other fish present in the scene. The abundance of other fishes was considered as a covariate in the data analysis. White points denote the bait. The fishing gear was maintained vertically by a float located at the upper end of the main line.

To test our hypothesis, a robust measure of fish naïveté in the field was derived. Underwater video recording has previously been used to observe the behaviour of wild marine fish around baited hooks [62,63], and the recent miniaturization of recording devices has opened new opportunities for testing novel behavioural hypotheses on naturally behaving fish in the wild [64]. In this study, the latency time (LT), observed via underwater video recording, of a fish when taking a natural bait offered on a dysfunctional hook (where the sharp end was removed) was used as a surrogate of naïveté as in our previous work [30]. To present the bait we used a customized device (Fig 2), which was designed to emulate the behaviour of natural baits on small-sized hooks [54]. The experimental design was similar to the design proposed by Carter et al. [65] for analysing the behaviour of the lizard *Agama planiceps* near traps and it has been developed and calibrated to angling conditions in our study area [30]. In our case, five hooks (size: 4; gape: 7.30 ± 0.03 mm), were mounted on a 0.35 mm monofilament nylon main line and were baited with similar-sized pieces of thawed shrimp, *Penaeus vannamei* (Fig 2). Thawed shrimp is the most common bait used by local anglers. We then recorded a potential capture by observing the intake of the experimental hooks by the focal fish.

The experimental protocol consisted of jointly deploying three different underwater cameras at each of the 54 sampling points. The three devices were identical and were deployed at a minimum distance of 50 m to each other to ensure that one device would not interfere with fish behaviour on a nearby device. The cameras continuously recorded in full high definition mode (30 fps and 1080 p) over the seagrass for a duration of 10 min. Due to logistical constraints, it was impossible to sample all 54 sampling points in a single day, and we therefore stratified the sampling over four days (sampling time from 9:00 AM to 1:00 PM). A different number of randomly selected sampling points were visited each day, and the date of the sampling was considered as a random factor in statistical analyses (see below). Sampling points were visited following a fully randomized sequence to prevent confounding effects of the position of the sampling points, the sampling day or any environmental variable, including distance to the MPA boundary.

### Data analysis

LT was defined as the time (in seconds) elapsed between the moment that a fish of one of the four species included in the study entered the scene until it tried to ingest the bait, which was a behaviour clearly recognized in the videos as the fish quickly shakes its head in an attempt to remove the bait from the hook (Fig 2). A survival analysis, which is an analysis designed to investigate the factors affecting the time at which a particular event (here taking the bait) occurs, was used to test the hypothesis [66]. In the present case, the response variable was LT (in seconds), which is a surrogate of fish naïveté/vulnerability and ultimately of fish survival probability. Not all of the observed fish ingested the bait within the duration of an experimental trial. As LT is unknown for those fish, the partially missing data were considered as right-censored observations [66]. A specific likelihood function (Cox regression) has been developed to account for this type of right-censored data [67], which was employed in this study.

We used the Cox regression to describe the probability of non-capture against a set of potential explanatory variables, including the variable of interest (i.e., distance to the boundary of the MPA). Multiple possible confounding variables were assessed and subsequently controlled in the modelling process. Although all of the experimental trials were conducted in seagrass meadows, we also considered the habitat as a covariate (e.g., presence of sand, rocks or mud in the seagrass), which generated multiple combinations of habitat types. We reduced the dimensionality of the habitat matrix using a principal component analysis (PCA, [68]) and used the scores of the two first axes as a covariate for controlling the habitat characteristics similar to Alós et al. [30]. Using the video footage, we also quantified the number of competitors present by counting the number of fish belonging to either the same or other species located within the scene at the moment that a focal fish appeared (Fig 2).

The full statistical model included five fixed variables (i.e., species, distance to the MPA, interaction of species × distance to the MPA, habitat type and the abundance of other fishes) and a random factor (i.e., the date effect). According to our working hypothesis we expected the decrease in LT with increasing proximity to the MPA for the intrinsically most catchable species (*S*. *scriba*) to be the greatest and the decrease in LT with increasing proximity to the MPA for the intrinsically less catchable species (*C*. *julis* and *D*. *annularis*) to be gradual or non-existent. The LT for the unharvested control species (*C*. *chromis*) should remain stable across all sampling points. Therefore, the effect of interest for testing our hypothesis was the interaction effect of species × distance to the MPA. We used the *coxph* function from the *survival* library of the R package (developed by T. Therneau and T. Lumleyat; http://cran.r-project.org/web/packages/survival/survival) to estimate the model parameters of the minimal adequate model. Stepwise model selection was completed using Akaike Information Criterion (ΔAIC) through the function *step* of the *survival* library, and the predicted survivorship probability at different times was plotted using the function *survfit* from the same library. To improve the visualization of the results, we categorized the distances to the MPA as either within, close or far based on negative distances to the boundary (i.e. within) and median based splitting of distance to define the categories close and far. Accordingly, sampling points within the MPA were located at -729.5 ± 253.5 m (average ± standard deviation), close points were located at 3,669 ± 1,771.4 m (average ± standard deviation), and far points were located at 8,254.8 ± 1,144 m. All analyses were run in a custom script of the R package (version, R-3.0.1, [69]).

## Results

**Fig 3:**
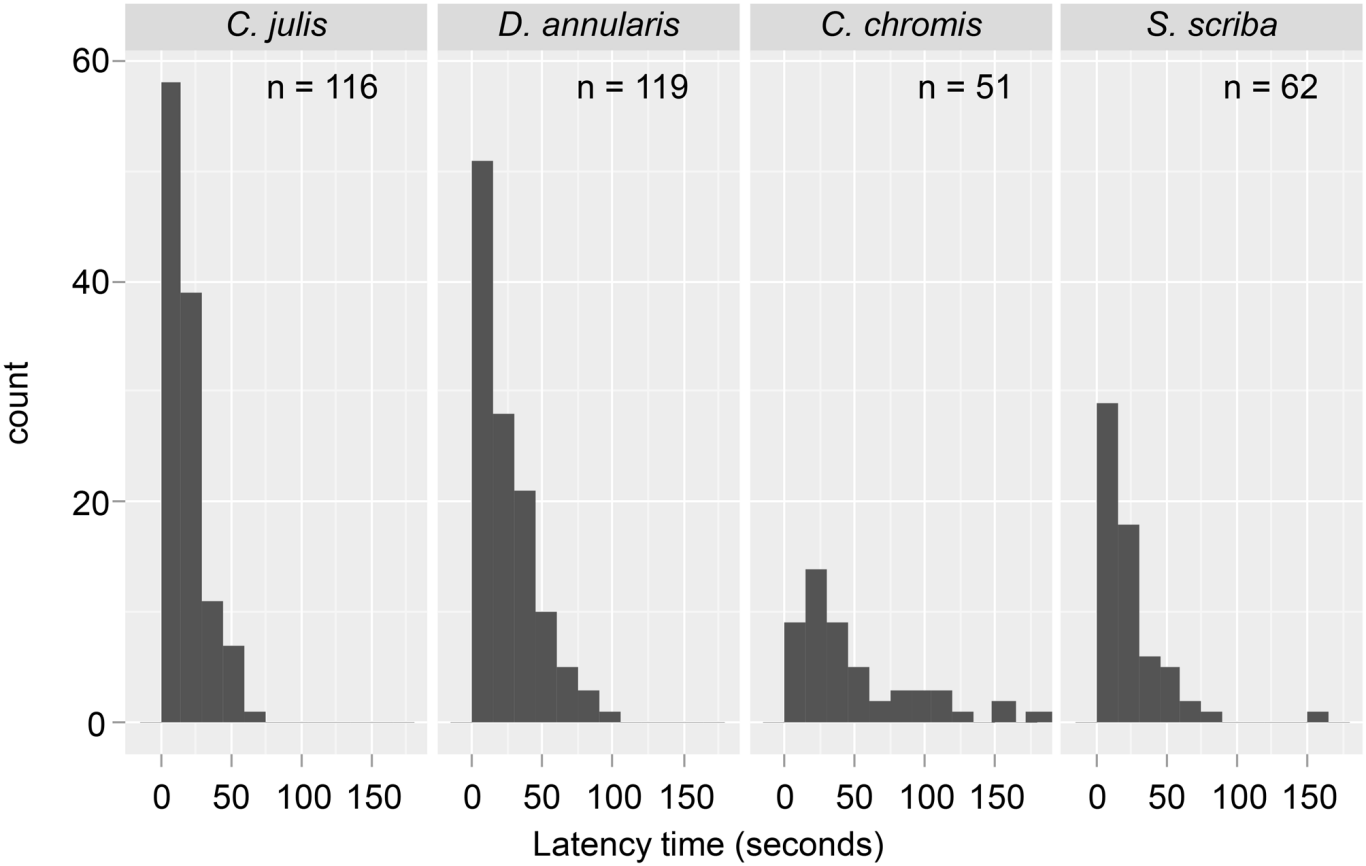
Histogram of the latency times (in seconds) and sample size of the four species considered in this study.

The LT from 348 individual fish were recorded after visualizing 1,620 minutes of video images corresponding to three 10 minute long video replicates, obtained at the 54 locations (Fig 3). For fish that took the bait, LT varied from 1 to 181 s and was on average (± s.d.) 23.2 ± 25 s, 18.5 ± 13.6 s, 25.2 ± 20 s and 50.2 ± 43.1 s for *S*. *scriba*, *C*. *julis*, *D*. *annularis* and *C*. *chromis*, respectively (see Fig 3 for a distribution of LT values of each of the four species). Relative to the uncatchable control species *C*. *chromis* as a baseline, the Cox regression coefficients for the LT of the other three species were significantly smaller (Table 1). The difference in the Cox regression coefficients with respect the control species was especially large for *S*. *scriba*, suggesting a high catchability of this species and intermediate levels of intrinsic catchability for *C*. *julis* and *D*. *annularis* as expected (Table 1). Neither the distance to the MPA boundary nor the density of other fish affected LT as main effects (Table 1). However, in support of the working hypothesis we found a significantly decrease of LT as distance to the MPA increased for the intrinsically highly catchable *S*. *scriba*, and only a non-significant decreasing trend of the LT for the two intrinsically less catchable *D*. *annularis* and *C*. *julis* (Table 1). The statistical support for our working hypothesis is that the interaction of *S*. *scriba* × distance to the MPA was significant (Table 1).

**Table 1:** Results of the survival analysis (Cox regression model). The effects on LT (seconds) of the explanatory variables, species, distance to the MPA and their interaction and the density of fish were retained by the AIC-based model selection. The regression coefficients (coef) of the logarithm of the hazard ratio and its standard error (s.e.), the exponent of the coefficient for interpretation, the z-value and p-value of the minimal adequate Cox regression model are shown.

Variable tested	coef	exp(coef)	s.e.(coef)	z-value	Pr(>|z|)
*C*. *julis*	3.5360	34.34	0.9145	3.87	<0.001
*D*. *annularis*	2.3700	10.70	0.9192	2.58	<0.05
*S*. *scriba*	4.3680	78.90	0.9176	4.76	<0.001
Distance (m)	-0.0001	0.9999	0.0001	-0.55	0.581
Other fish	0.0091	1.0090	0.0056	1.63	0.104
*C*. *julis* × D	0.0001	1.0000	0.0001	0.46	0.648
*D*. *annularis* × D	0.0001	1.0000	0.0001	1.01	0.314
*S*. *scriba* × D	-0.0003	0.9997	0.0002	-2.00	<0.05

Concordance = 0.724 (s.e. = 0.026). R-square = 0.331 (max possible = 0.991).

**Fig 4:**
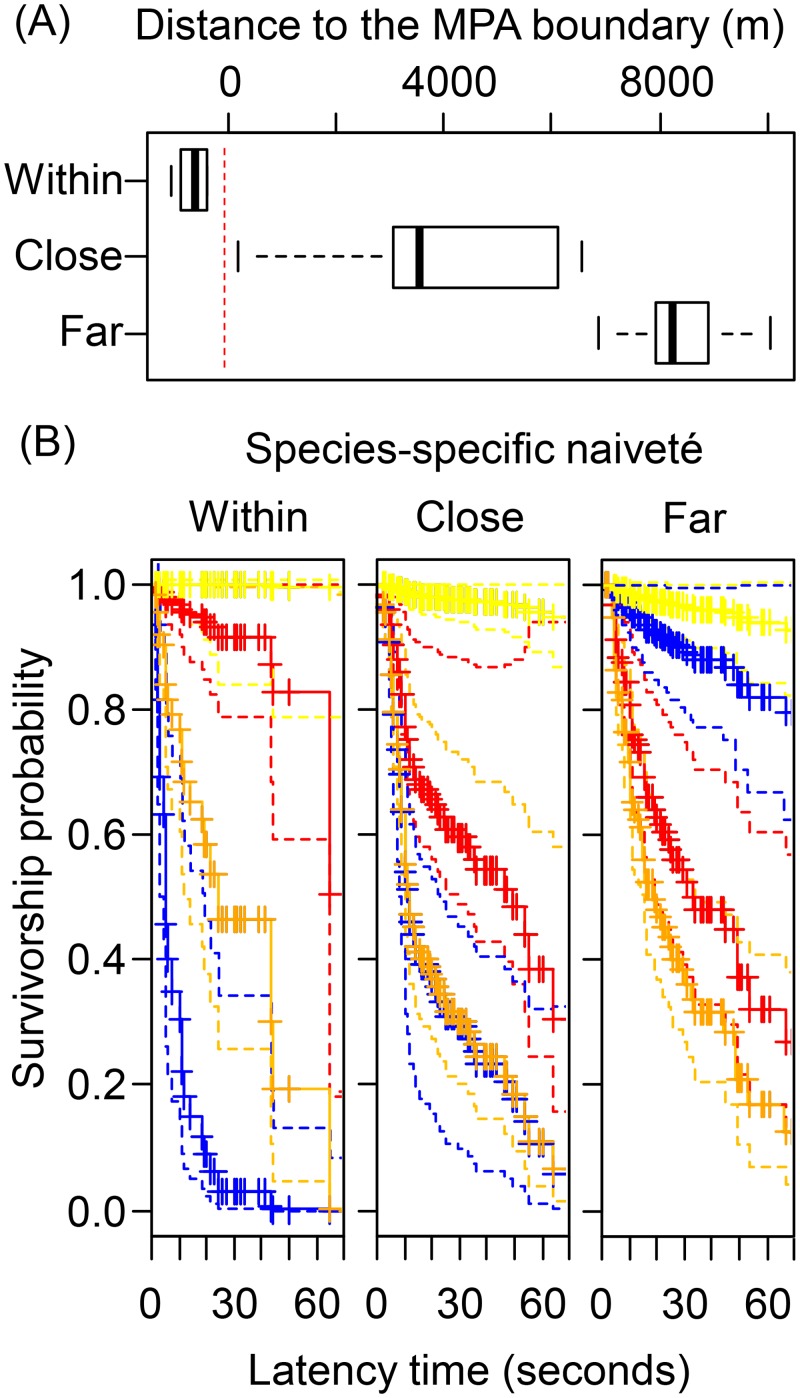
Predicted survivorship from the Cox regression. The panel (A) shows a box-plot of the distribution of distances to the boundary for sampling points within (up), close (mid) and far (lower) to the MPA. The vertical red dashed line indicates the boundary of the MPA. The panel (B) shows the predicted survivorship probability (y-axes) as a function of latency time (LT) (x-axes, in seconds) for the four species considered: *C*. *julis* (orange), *D*. *annularis* (red), *C*. *chromis* (yellow) and *S*. *scriba* (blue). The lower-left, lower-mid and the lower-right panels correspond to sampling point locations within, close and far to the MPA, respectively,. Solid lines indicate the expected survivorship probability and dashed lines the 5% confidence intervals. Note that *S*. *scriba* shows different survivorship (~naïveté) pattern depending on the distance to the boundary of the marine protected area (MPA).

The survivorship plots aimed to visually represent the effects of the distance of the MPA on the LT of the various species (Fig 4). These plots were consistent with the results just described that *S*. *scriba* was the most naïve species. This is indicated by the smallest LT and hence low theoretical survival probability within and close to the MPA (Fig 4). The corresponding values of the LT for *C*. *julis* and *D*. *annularis* close to the MPA were also small; most of the fish would have been captured within the first minute of bait exposition (Fig 4). Conversely, the LT of *C*. *chromis* was very long, suggesting that this species remained uncatchable to angling and that the survivorship probability was close to 1 (full survival) even near the MPA (Fig 4). The survivorship plot that corresponded to the sampling points far from the MPA showed similar trends for all species except for *S*. *scriba* that scored second in relation to LT, just after the largely uncatchable *C*. *chromis* (Fig 4). This indicated a low naïveté level for *S*. *scriba* in locations far away from the MPA (Fig 4). In fact, at far locations only 20% of *S*. *scriba* in vicinity to baited hooks may be expected to be captured, based on the results, while in areas within and close to the MPA almost 100% of the fish may be hooked (Fig 4). In far distance sites, *C*. *julis* and *D*. *annularis* again showed intermediate naïveté values between the highly catchable *S*. *scriba* and the nearly uncatchable *C*. *chromis* (Fig 4).

## Discussion

We found empirical evidence that fish naïveté to recreational angling gear decreases with increasing distance from the boundary outside of a fully protected MPA. A significant relationship between the LT to ingest a bait and the distance to the boundary was, however, only evident in the intrinsically highly catchable species (i.e., *S*. *scriba*). For this species, we found a gradient from high naïveté (short LT) at sites within and close to the MPA, to low naïveté (larger LT) at far sites. Conversely, the other two intrinsically less catchable omnivorous species and the largely unharvested *C*. *chromis* showed similar LT, irrespective of the distance to the MPA. These results suggest that naïve individuals of intrinsically highly catchable species are prone to be quickly harvested at sites located near an MPA whenever fishers “fish along the line” of MPA boundaries [70]. This fact emphasizes the importance of considering the spatial behavioural dimension of fish when predicting the outcomes of MPAs because the effective contribution of spillover may be smaller than anticipated for highly catchable species such as *S*. *scriba*.

Our results are consistent with recently reported findings which suggest that MPAs may spread naïve fish of some species, but not others [22]. A theoretically conceivable “spillover of naive fish” from MPAs for some species is expected to be mechanistically caused by the spillover of behaviourally vulnerable fish [32]. Such effects, if widespread, would be very positive from the fisher’s perspective by increasing catch rates outside the no-take areas, thereby enhancing the benefits of MPAs for fisheries [10]. However, our results suggest that in the case of passively operated recreational angling gears, a gradient-based pattern of fish naïveté should only be expected in the intrinsically highly catchable species. It is also conceivable that the benefits of a potential spillover of naïve fish would only be experienced at very short distance to the MPA, particularly when fishing intensities are high.

In addition, the species-specific nature of distance-based naïveté found in our work and in related studies for spearfishing [32] suggests that many species will not show a vulnerability gradient at all but will have consistently low levels of vulnerability in protected and unprotected sites (see also [30]). One of the main predictors of intrinsic catchability at the species level is the position in the food web. Species located at higher trophic levels are not only more intensively targeted by anglers [36], but they may also be behaviourally characterized by very high vulnerability to angling, due to greater mobility or larger aggression levels compared to low trophic level fishes [30,71]. In the *P*. *oceanica* seagrass meadows ecosystem, *S*. *scriba* (the carnivorous species) is one of the species located at high trophic levels [60]. By contrast, *D*. *annularis*, *C*. *julis* and especially *C*. *chromis* are species positioned lower in the food web because they mainly forage on sessile, benthic, or small pelagic food sources [60]. Although our sample size for the carnivorous feeding guild is essentially restricted to one species, we tentatively suggest that exporting naïve individuals outside of MPAs could be more pronounced in carnivorous species and that these species should be intrinsically more catchable to recreational angling. Testing this hypothesis with a larger sample of feeding guilds constitutes an important research avenue for the future.

The mechanisms behind the enhanced naïveté of *S*. *scriba* living within or near MPAs are twofold, because it may involve an evolutionary response, a learning-based adaptation within the realm of behavioural plasticity, or both [72]. Any behaviourally-selective fishing will enhance the survival probability of genetically determined, low vulnerability phenotypes outside protected areas, which will reduce the naïveté of fish to fishing gears and also increase their average timidity levels as an evolutionary response [30,34,58]. Previous research in other species has shown that vulnerability has a genetic basis in largemouth bass (*Micropterus salmoides*) and carp [39,42]. It is hence possible that evolutionary changes may have played a role in explaining the reduction in vulnerability in *S*. *scriba*. The second mechanism focuses on the ability of fish to modify their behaviour after learning from previous encounters with predators (which includes fishing gear) [39]. Deviations from normal behaviour that reduce hooking propensity have been well documented in range of freshwater recreational fisheries [39,44,47,49,73]. There is evidence that such learning depends on the foraging mode, where it is more pronounced in omnivorous fishes [46]. However, a reduction in vulnerability to angling has also been reported for strictly piscivorous pike exposed to heavy fishing pressure [48,74], particularly when exposed to artificial lures [47]. Therefore, the gear-avoidance observed in *S*. *scriba* outside the MPA might also be caused by learning from previous hooking events or by observing other individuals being harvested [30]. Collectively, fish inhabiting protected areas should eventually display an increased naïveté to fishing gears because of these two mechanisms—reduced strengths of selection by fishing gear and learning in areas that are safe from human-induced predation risk. However, whether this increased naïveté is caused by an evolutionary change or (plastic) learning can not be unequivocally answered with our experimental design. Further research is needed to investigate the main cause that generates the patterns we observed here for *S*. *scriba*. One way to do this could be through rearing individuals from different sites under controlled conditions in laboratory environments, followed by the measurement of the vulnerability of the offspring to separate genetic from plastic responses.

Naïveté of fish measured through flight initiation distance in the case of spearfishing or LT in the case of recreational angling with natural baits may be interpreted as a measure of boldness [30,75], which is defined as risk-sensitive foraging [76]. Human disturbances and past fishing can be conceptualized as a form of predation risk [32,74,77]. Hence, a fish’s readiness to accept the closeness of a diving human and the attraction to a baited hook in the presence of noisy angling boats may represent boldness as a personality trait. Unfortunately, our measure of LT is not sufficient for defining it as a personality trait because repeatability and the consistency of individual differences across time or contexts was not assessed, which is a precondition to interpret a particular behaviour as a personality trait [78]. However, previous research in *S*. *scriba* [30,58] has reported that individuals living in low exploitation sites or inside an MPA forage more intensively in the presence of risk than those located outside an MPA. Boldness is related to an individual’s foraging capability in the presence of risk and, thus may be related to individual growth and productivity [79]. As a result, the individuals inhabiting sites close to or inside MPAs should be more productive with regards to biomass production than shy individuals outside of an MPA unless density-dependent food limitation associated with larger overall biomasses in an MPA areas overrides any behaviourally-based increased foraging propensity. We have previously shown that *S*. *scriba* inhabiting an MPA attain larger final body sizes than those inhabiting exploited areas [58]. This could be a either a life-history adaptation to size-selective harvesting and high fishing pressure outside of the MPA or link between boldness and productivity, or both.

The increased vulnerability (or decreased naïveté) to the recreational fishing gear with increasing proximity to the MPA observed in *S*. *scriba* could have implications for the relationship between catch rates and fish abundance [30]. Hilborn and Walters [80] developed the idea of hyperdepletion as a mechanism that would explain why catch rates decline more rapidly than fish abundance. Whenever fish become less vulnerable in response to increasing exploitation, hyperdepletion may occur unless effort by more skilled anglers or schooling overrides any behaviourally-based decoupling of the catch rate and abundance, which in turn may lead to hyperstable catch rates [81]. Although hyperdepletion might occur more commonly in intrinsically highly catchable species than it was previously believed, few mechanistic studies have described the possible behavioural processes that can cause this outcome [30]. Our results provide a possible explanation for the mismatch between catch rates and abundance in selected species, and suggests that a decline of the catch rate with respect to distance to the MPA is possible in some species such as *S*. *scriba* [30]. Hence, spatial variation in fishery-dependent catch rate data has to be expected and accounted for in fishery-dependent stock assessments that collect data over wide spatial scales. More studies such as the present one are needed to fully understand the magnitude of such a sampling bias and its implications for stock assessments. Before this research becomes available, one should be careful when inferring spillover trends from catch-rate dependent data collected from recreational angling, spearfishing, trapping or longlining because the reduced naïveté with increasing distance to the fully protected MPA is aloso expected to occur for some species.

We conclude that spillover of naïve individuals from MPAs previously reported for spearguns is expected to occur also in some species targeted by recreational angling. However, the exportation of naïve individuals and hence biomass is not generalizable across species. This effect should be particularly evident in species that are intrinsically more catchable to fishing, such as carnivorous species. The spillover of naïve individuals for these highly catchable species may also be less relevant than expected when fishing pressure at the boundaries of a MPA is high. Any local enhancement of catch rates can nevertheless provide a direct benefit to recreational fisheries because catch rates positively influence angler satisfaction in recreational fisheries [82,83]. In addition, any systematic changes in vulnerability will undermine a stock assessment based on fishery-dependent data as it would result in hyperdepletion where catch rates are decoupled from the underlying abundance [30]. We therefore recommend that to avoid sampling bias, a combination of fishery-dependent and independent data are best for assessing the abundance of exploited fishes. Fish behaviour plays a key role in determining and modulating the impact of fishing on wild populations [84], and the incorporation of the behavioural dimension on the spillover of a MPA should, therefore, improve the predicted benefits of MPAs at larger spatial and temporal scales.
